# Backpack PCR: A point-of-collection diagnostic platform for the rapid detection of Brugia parasites in mosquitoes

**DOI:** 10.1371/journal.pntd.0006962

**Published:** 2018-11-21

**Authors:** Weam I. Zaky, Francesca R. Tomaino, Nils Pilotte, Sandra J. Laney, Steven A. Williams

**Affiliations:** 1 Department of Biological Sciences, Smith College, Northampton, Massachusetts, United States of America; 2 Molecular and Cellular Biology Program, University of Massachusetts, Amherst, Massachusetts, United States of America; 3 The Bill and Melinda Gates Foundation, Seattle, Washington, United States of America; QIMR Berghofer Medical Research Institute, AUSTRALIA

## Abstract

**Background:**

Currently, molecular xenomonitoring efforts for lymphatic filariasis rely on PCR or real-time PCR-based detection of *Brugia malayi*, *Brugia timori* and *Wuchereria bancrofti* in mosquito vectors. Most commonly, extraction of DNA from mosquitoes is performed using silica column-based technologies. However, such extractions are both time consuming and costly, and the diagnostic testing which follows typically requires expensive thermal cyclers or real-time PCR instruments. These expenses present significant challenges for laboratories in many endemic areas. Accordingly, in such locations, there exists a need for inexpensive, equipment-minimizing diagnostic options that can be transported to the field and implemented in minimal resource settings. Here we present a novel diagnostic approach for molecular xenomonitoring of filarial parasites in mosquitoes that uses a rapid, NaOH-based DNA extraction methodology coupled with a portable, battery powered PCR platform and a test strip-based DNA detection assay. While the research reported here serves as a proof-of-concept for the backpack PCR methodology for the detection of filarial parasites in mosquitoes, the platform should be easily adaptable to the detection of *W*. *bancrofti* and other mosquito-transmitted pathogens.

**Methodology/Principal findings:**

Through comparisons with standard silica column-based DNA extraction techniques, we evaluated the performance of a rapid, NaOH-based methodology for the extraction of total DNA from pools of parasite-spiked vector mosquitoes. We also compared our novel test strip-based detection assay to real-time PCR and conventional PCR coupled with gel electrophoresis, and demonstrated that this method provides sensitive and genus-specific detection of parasite DNA from extracted mosquito pools. Finally, by comparing laboratory-based thermal cycling with a field-friendly miniaturized PCR approach, we have demonstrated the potential for the point-of-collection-based use of this entire diagnostic platform that is compact enough to fit into a small backpack.

**Conclusions/Significance:**

Because this point-of-collection diagnostic platform eliminates reliance on expensive and bulky instrumentation without compromising sensitivity or specificity of detection, it provides an alternative to cost-prohibitive column-dependent DNA extractions that are typically coupled to detection methodologies requiring advanced laboratory infrastructure. In doing so, this field-ready system should increase the feasibility of molecular xenomonitoring within *B*. *malayi*-endemic locations. Of greater importance, this backpack PCR system also provides the proof-of-concept framework for the development of a parallel assay for the detection of *W*. *bancrofti*.

## Author summary

Molecular xenomonitoring has demonstrated significant potential as a non-invasive means of providing reliable surveillance for the presence of lymphatic filariasis (LF)-causing parasites. Given the continuing successes of global mass drug administration efforts, the need for such non-invasive surveillance techniques is expanding. However, considering the significant infrastructural demands which such surveillance requires, the development of simplified surveillance methodologies will be fundamental to future programmatic implementation efforts. Accordingly, we have developed a novel, simplified diagnostic platform for point-of-collection-based detection of the LF-causing parasite, *Brugia malayi* in pools of mosquitoes. By coupling a rapid and inexpensive DNA extraction methodology with a field-friendly amplification platform and test strip-based detection assay, this backpack PCR system eliminates the need for expensive instrumentation and laboratory-based infrastructure. Furthermore, adaptation of the platform described here will allow for the straightforward and rapid development of a parallel assay for the detection of *Wuchereria bancrofti*, facilitating the increased use of xenomonitoring and enabling mosquito surveillance efforts in regions lacking sophisticated laboratory infrastructure.

## Introduction

Lymphatic filariasis (LF) is a disfiguring and disabling tropical disease caused by parasitic filarial nematodes. It is estimated that more than 120 million people currently suffer from this mosquito-borne infection, with approximately 90% of the global LF burden caused by *Wuchereria bancrofti* and the remaining 10% caused by the parasites *Brugia malayi* and *Brugia timori* [1–3]. Despite this significant burden of disease, the World Health Organization has targeted LF for elimination by the year 2020 [4–7], and accordingly, the Global Programme to Eliminate Lymphatic Filariasis (GPELF) has implemented mass drug administration (MDA) programs in most endemic countries in order to interrupt disease transmission and reduce infection rates.

An important component of LF elimination efforts is monitoring changing infection and exposure rates over the course of yearly MDAs in order to evaluate programmatic success and determine when treatment can be stopped [8–11]. Following the cessation of MDA, similar monitoring is required to ensure that infection recrudescence has not occurred and is unlikely to occur going forward [12]. While human blood sampling is currently the standard procedure for monitoring these rates [13–16], PCR-based detection of parasite DNA in insect vectors, termed molecular xenomonitoring (MX), is an effective and non-invasive alternative, capable of indirectly measuring parasite burden within endemic locations [17–27]. Given this potential, the World Health Organization has championed the development of novel methodologies capable of increasing the practicality of this approach to disease surveillance [28–29], since implementation of currently available MX techniques are expensive in terms of both reagents and infrastructure requirements.

Currently, field-adaptable loop-mediated isothermal amplification (LAMP) assays exist for the detection of both *W*. *bancrofti* [30] and *Brugia* parasites [31], and a helicase-dependent isothermal amplification (HDA) assay exists for the detection of *B*. *malayi* DNA in human blood samples [32]. However, widespread programmatic implementation of such assays has not occurred. Limited use likely stems primarily from insufficient large-scale comparative evaluation efforts. Concerns regarding detection ambiguities and uncertainties may also contribute. When running LAMP assays detection of positive samples relies on the visualization of either sample turbidity or sample fluorescence [31]. As such, results are not “presence” or “absence”-based, but rather occur on a spectrum. This potentially raises concerns regarding susceptibility to technician bias, fatigue, or interpretation. Equally problematic, while HDA assays typically rely on agarose gel electrophoresis, a proven means of effective target detection, such techniques are prone to sample contamination [33], and are difficult to perform in a field setting. In contrast, the field-friendly, “Backpack PCR” platform we describe here utilizes test strip-based DNA detection methods. Such technology partners the advantages of standardizing assay readouts with the capacity to minimize laboratory equipment needs. Furthermore, given the ongoing work towards the development of automated card reader technologies [34], the consistency of results will likely continue to improve. For these reasons, a number of assays conducted in conjunction with the Global Programme to Eliminate Lymphatic Filariasis and other tropical disease control and elimination programs [35] make use of strip or card-based detection techniques [36–39].

Here we describe the development of a novel diagnostic platform, which we have termed “Backpack PCR”, coupling a rapid mosquito DNA extraction procedure with the test strip-based detection of *B*. *malayi* DNA. This assay amplifies a 132 base pair region of the non-coding *HhaI* repetitive DNA sequence element, selected as the target due to its high copy number and proven reliability in previously described molecular diagnostic assays [32, 40–45]. Following a resource-minimizing NaOH-based DNA extraction procedure, amplification occurs using a field-friendly, miniaturized PCR technology. Parasite DNA-derived amplicons are then detected using a test-strip-based methodology, enabling point-of-collection-based sample processing. While acknowledging the potential utility of backpack PCR as a tool in *Brugia-*endemic locations, the primary contribution of this work is likely its service as a proof-of-concept study for the parallel development of a similar assay for the detection of *W*. *bancrofti*. Such development is currently underway.

## Materials and methods

### Preparation of mosquito pools

While *B*. *malayi*-infected mosquitoes can be successfully reared in the laboratory, due to the possible failure of individual mosquitoes to ingest microfilariae when taking a blood meal, the definitive assumption cannot be made that all exposed mosquitoes actually harbor parasites. For this reason, positive mosquito pools were prepared by spiking groups of uninfected, laboratory reared *Aedes aegypti* mosquitoes (Oxitec Ltd., Abingdon, UK) with a single *B*. *malayi* infective-staged larva (L3) (Filarial Research Reagent Resource Center [FR3], Athens, GA). Uninfected *A*. *aegypti* mosquito pools were also prepared to serve as experimental controls. Positive pools containing 5, 10, 15, 20, and 25 uninfected mosquitoes were prepared by adding a single L3 larva to each pool. Twenty positive pools of each pool size were prepared, while two negative control pools, containing only uninfected mosquitoes with no added L3 larva, were also prepared for each pool size. Thus, 22 pools total were prepared for each of the 5 pool sizes, resulting in 110 total pools.

### DNA extraction from pooled mosquitoes

Ten positive mosquito pools and 1 negative mosquito pool of each mosquito pool size (5, 10, 15, 20 and 25) were extracted using a modified version of a previously published, cost-effective, NaOH-based DNA extraction procedure [46]. Briefly, in a 1.7 ml microfuge tube, a sterile plastic micro-pestle (Axygen Scientific, Union City, CA) was used to grind each mosquito pool with 180 μl of 0.2 N NaOH for 3 min. The micro-pestle was then rinsed with an additional 180 μl of 0.2 N NaOH into the same 1.7 ml tube containing the ground mosquitoes to be sure all mosquito debris was removed from the pestle. A new, clean, sterile micro-pestle was used for each pool. Each tube was incubated at 75 °C for 10 min. 115.2 μl of 1 M Tris (pH = 8.0) and 364.8 μl of nuclease-free water were added to each sample and the tubes were thoroughly mixed using a vortex mixer for 10 sec. Samples were centrifuged at 10,000 x g for 3 min, and the supernatant, containing extracted DNA, was collected and transferred into a clean 1.7 ml microcentrifuge tube. Utilizing an additional volume of nuclease-free water, ten-fold dilutions of each sample were prepared to minimize the effects of PCR inhibitors on downstream amplification reactions. In parallel, ten positive and 1 negative mosquito pool of each mosquito pool size (5, 10, 15, 20 and 25) were also extracted utilizing the Qiagen DNeasy Blood & Tissue Kit (Qiagen, Hilden, Germany) in accordance with the previously described extraction protocol [43, 47–48].

### Real-Time PCR testing of DNA extracts

Real-time PCR was used to determine whether or not each pool of mosquito DNA, extracted by either the NaOH-based or Qiagen-based method, contained *B*. *malayi* DNA. For testing purposes, the real-time PCR assay developed by Rao, *et al*. was considered to be the gold standard for parasite detection [43], and it was assumed that every pool containing *B*. *malayi* DNA would result in positive detection using this assay. All real-time PCR reactions were carried out in triplicate using the previously described primers, probe, reaction mixture, volume, and cycling conditions [43]. Thermal cycling was performed using the StepOnePlus Real-Time PCR System (Applied Biosystems, Foster City, CA). Results were considered to be positive when amplification occurred with a mean Ct value of less than 38.

### Amplification of *B*. *malayi* DNA utilizing standard and miniPCR platforms

A comparison of amplification efficiency between a standard thermal cycler (Veriti Thermal Cycler, Applied Biosystems, Foster City, CA) and the field-friendly miniPCR platform (Amplyus LLC, Cambridge, MA), was conducted using conventional PCR-based amplification of *B*. *malayi* DNA. The reactions were carried out using the Phire Hot Start II enzyme (Thermo Fisher Scientific, Waltham, MA) with the previously described real-time PCR primer set [43], modified to contain a biotin tag on the reverse primer (Fwd: 5’—GCAATATACCGACCAGCAC—3’ / Rev: 5’—Biotin-ACATTAGACAAGGAAATTGGTT—3’). Reactions were performed in 20 μl total volumes containing 100 nM concentrations of each primer, 0.5 μl of enzyme, 5 μl of 5X reaction buffer, 0.5 μl of 10 mM dNTPs, and 1 μl of a 10-fold dilution of the DNA extraction product. Parallel amplification reactions were performed using both a standard thermal cycler, (Veriti Thermal Cycler, Applied Biosystems), and the miniPCR instrument (Amplyus LLC, Cambridge, MA). Cycling conditions consisted of an initial 30 sec hold at 95 °C, followed by 35 cycles of 30 sec at 95 °C, 40 sec at 55 °C, and 1 min at 72 °C. Following cycling, a final 5 min extension step at 72 °C was performed.

### Test strip-based detection of amplification products

#### Labeling of the amplified product

**Fig 1:**
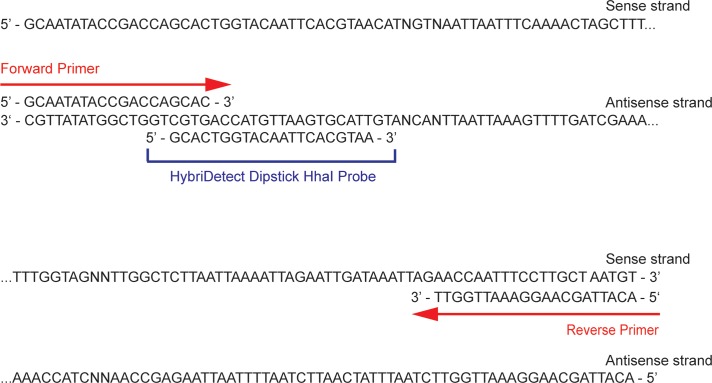
*B*. *malayi* HhaI repeat DNA sequence used for test strip-based detection. The sequence of *B*. *malayi* DNA that is amplified from the *HhaI* repetitive target is illustrated. Both the forward and reverse primer sites, as well as the location of the hybridization probe are indicated.

HybriDetect dipsticks (Milenia Biotec, Giessen, Germany) were used for the development of the detection assay. Detection of the amplified product with HybriDetect test strips requires that the DNA product be labeled with both a biotin tag (in order to bind to the streptavidin band of the test strip) and a FAM tag (in order to bind to the gold-labeled anti-fluorescein antibodies that result in a visible band for detection). Biotin was incorporated into the product of the PCR reaction by labeling the reverse primer as described above. FAM was incorporated into the product using a 5’ FAM-labeled sequence-specific probe that was designed using Primer Express 3.0.1 software (ThermoFisher Scientific) to be complementary to the strand of DNA tagged with the biotin label (Fig 1). Both the biotin-labeled primer and the FAM-labeled probe (5’—FAM-GCACTGGTACAATTCACGTAA—3’) were ordered from Integrated DNA Technologies (Coralville, IA).

#### Probe hybridization and test strip detection

**Fig 2:**
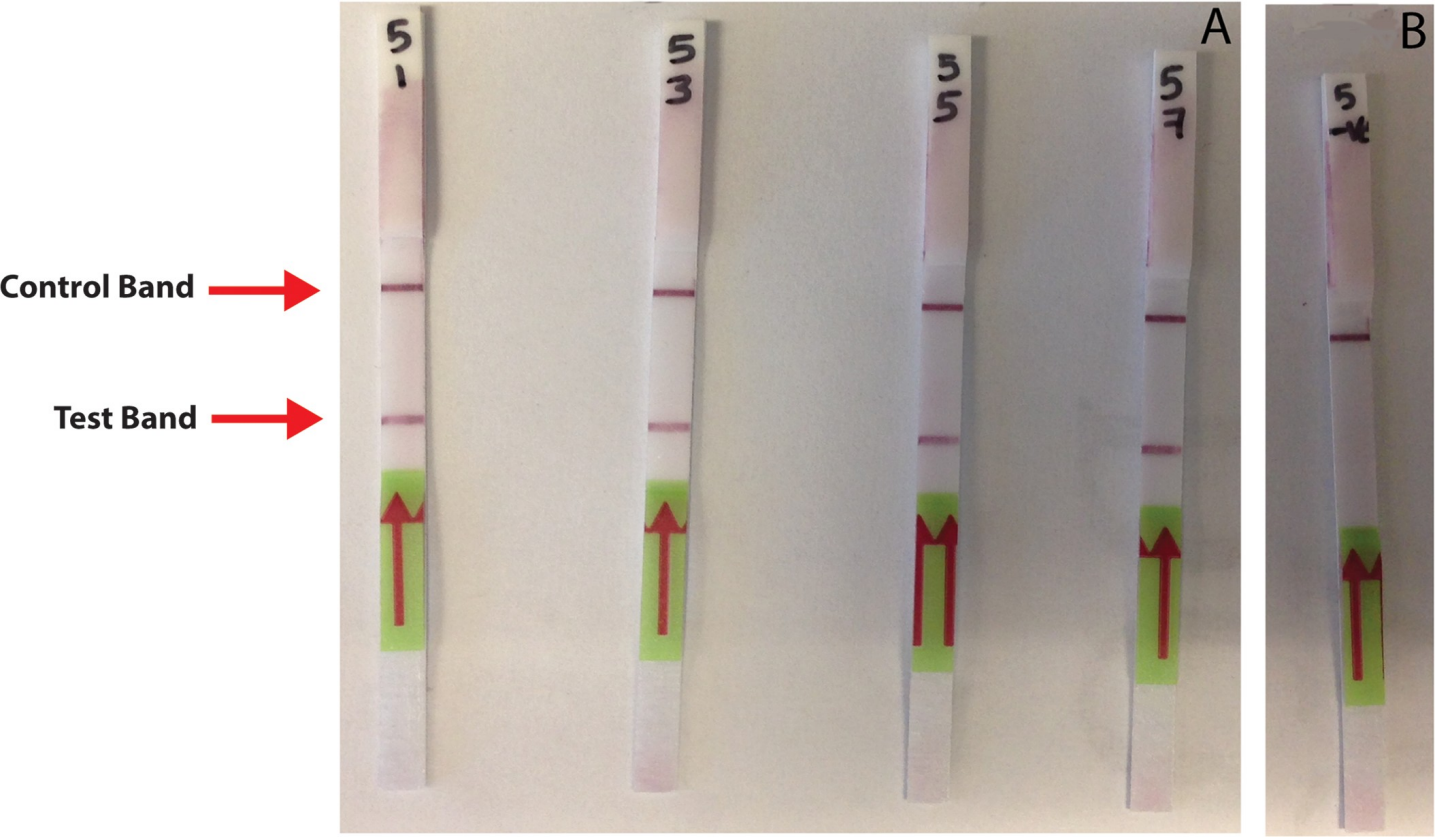
Test strip-based detection of amplification products generated by miniPCR. A dark purple control band is visible near the top of each test strip, indicating the proper functioning of the dipsticks. (A) Only samples containing PCR amplification products generated from *B*. *malayi* DNA produce a visible band (test band) below the location of the control band. (B) Negative control reactions do not display visible accumulation of tagged amplification product at the location of the test band, indicating that parasite DNA was not present in these samples.

Following conventional PCR amplification of each sample using both the standard PCR and the miniPCR instruments in parallel, the 5’ FAM-labeled probe was hybridized to each resulting amplicon creating a double-stranded DNA molecule containing both a primer-derived biotin tag and a probe-derived FAM label. This hybridization reaction was performed in accordance with the manufacturer’s published protocol. After hybridization, the full volume of the reaction mixture was added to 50 μl of HybriDetect Assay Buffer (Tris-buffered saline), and a HybriDetect dipstick was placed into each sample tube and allowed to incubate at room temperature for 15 min. Following incubation, the dipstick was removed and visually inspected for the presence of a control band and a positive test band. A positive test band appears as a result of the biotin-labeled PCR product binding to a horizontal streptavidin test band as the sample travels up the strip by capillary action. Polyclonal (rabbit) anti-FITC antibodies, each conjugated to a gold particle, then bind to the FAM-labeled product, and the congregation of gold particles at the streptavidin test line causes a visible accumulation of color that is indicative of a positive result. A control band of anti-rabbit antibodies located above the test band also becomes visible as the sample travels along the length of the dipstick. The presence of this band is used to confirm the proper function of the test strip (Fig 2).

#### Comparative assay sensitivity testing

Analysis of the DNA extracted from spiked mosquito pools prepared as described above was used to assess the sensitivity of the test strip-based detection technique. For all tested samples, test strip results were compared with results of both the real-time PCR and the conventional PCR coupled with agarose gel electrophoresis. All conventional PCR reactions were performed using both the standard PCR and the miniPCR instruments.

#### Assay specificity testing

To verify the specificity of the test strip-based detection platform, genomic DNA samples isolated from *Dirofilaria immitis*, *Mansonella perstans*, *Wuchereria bancrofti*, *Brugia pahangi*, *Acanthocheilonema viteae*, *and Loa loa*, were used to measure off-target PCR amplification and strip test product detection. In order to confirm the presence of filarial-derived DNA in each sample, all gDNA isolates were first tested by conventional PCR using the previously published pan-filarial DIDR primer set [49]. All reactions were performed in 20 μl total volumes, utilizing 1 μl of gDNA template and 100 nM concentrations of both the forward and reverse DIDR primers. All remaining reaction components and cycling conditions were identical to those described above for the conventional PCR amplification of target DNA from *B*. *malayi*. Once the presence of PCR-amplifiable genomic DNA was verified, all DNA samples were then tested by real-time PCR utilizing the *Brugia-*specific HhaI assay [43]. The genomic DNA samples were also tested by conventional PCR for *B*. *malayi*, using the abovementioned real-time PCR assay primers and employing the same reaction recipes and conditions as described above. Detection of conventional PCR amplification products occurred using both test strips and agarose gel electrophoresis.

### Blind test for assay validation

Fifty pools of mosquitoes were prepared which contained either zero or one *B*. *malayi*-L3-staged larva in pools of 5, 10, 15, 20, or 25 uninfected mosquitoes. Pools were then coded blind to the technician performing the assay. All mosquito pools underwent DNA extraction using the NaOH-based method described above, and extracts underwent amplification using the miniPCR platform, a standard conventional PCR platform, and the StepOnePlus Real-Time PCR System. All conventional PCR products were visualized using both test strip-based detection and agarose gel electrophoresis. Real-time PCR results were used to confirm the presence or absence of parasite DNA within each sample. After recording all results, the samples were un-blinded, and results were assessed.

## Results

### Testing the efficiency of the rapid DNA NaOH extraction method

The efficiency of the rapid NaOH-based DNA extraction methodology was examined through comparative real-time PCR testing of NaOH and Qiagen column-extracted sample panels. Qiagen extractions resulted in positive parasite detection in 50/50 samples, while NaOH-based extractions resulted in positive parasite detection in 49/50 samples. The NaOH-based extraction method was sufficiently efficient to give consistent detection of a single *B*. *malayi* L3-staged worm in all pools of up to 20 uninfected mosquitoes and in 9/10 pools containing 25 mosquitoes (Table 1). All mosquito pools not containing an L3-staged parasite were negative by both methods.

**Table 1:** Comparative performance of NaOH-based and Qiagen column-based DNA extraction procedures for the real-time PCR-based detection of *B*. *malayi* in pools of mosquitoes.

Number of Samples	Pool Size (# of mosquitoes per pool)	Column-based Extraction Mean Ct Value (Ct range)	NaOH-based Extraction Mean Ct Value (Ct range)
**10**	5	32.6* (27.7–37.0)	31.2 (29.3–32.9)
**1 Uninfected**	5	Negative	Negative
**10**	10	31.0 (28.1–38.3)	31.6 (30.0–34.2)
**1 Uninfected**	10	Negative	Negative
**10**	15	30.9 (27.9–33.0)	32.6^ (28.0–38.6)
**1 Uninfected**	15	Negative	Negative
**10**	20	31.7 (26.7–35.6)	32.5 (28.8–36.6)
**1 Uninfected**	20	Negative	Negative
**10**	25	35.0 (31.7–39.4)	31.6^#^ (28.8–35.6)
**1 Uninfected**	25	Negative	Negative
**1 *B*. *malayi* +**	Positive Control	6.5	11.4
**1 NTC**	No Template Control	Negative	Negative

* Positive detection of one sample from this set occurred in 2 out of 3 replicates tested

^ Positive detection of one sample from this set occurred in 1 out of 3 replicates tested

# One sample in this set failed to allow for detection of parasite signal

### Sensitivity Testing of the miniPCR and Test Strip-Based Detection Methods

Following re-blinding of the panel of NaOH-extracted samples described above, conventional PCR-based amplification of all samples was conducted using both the miniPCR and the standard Veriti PCR instruments. Results were compared with those previously obtained by real-time PCR. For all samples tested, real-time PCR results agreed with the miniPCR results using both test strip-based detection and electrophoresis-based detection (Table 2).

**Table 2:** Comparison of the standard PCR-based amplification and miniPCR-based amplification of *B*. *malayi* DNA from mosquito pools coupled with real-time PCR-based detection.

Sample ID	Pool Size	Real-Time PCR Result (Ct Value)	Standard PCR Gel Result	miniPCR Gel Result	miniPCR Test Strip Result
**1**	5	30.7	+	+	+
**2**	5	32.2	+	+	+
**3**	5	29.3	+	+	+
**4**	5	32.9	+	+	+
**5**	5	30.4	+	+	+
**6**	5	31.9	+	+	+
**7**	5	32.3	+	+	+
**8**	5	29.5	+	+	+
**9**	5	30.9	+	+	+
**10**	5	31.6	+	+	+
**11 Uninfected**	5	-	-	-	-
**12**	10	30.2	+	+	+
**13**	10	31.2	+	+	+
**14**	10	31.2	+	+	+
**15**	10	34.2	+	+	+
**16**	10	31.3	+	+	+
**17**	10	31.2	+	+	+
**18**	10	30.0	+	+	+
**19**	10	32.4	+	+	+
**20**	10	32.9	+	+	+
**21**	10	31.3	+	+	+
**22 Uninfected**	10	-	-	-	-
**23**	15	33.9	+	+	+
**24**	15	31.7	+	+	+
**25**	15	35.0	+	+	+
**26**	15	30.8	+	+	+
**27**	15	31.9	+	+	+
**28**	15	34.1	+	+	+
**29**	15	28.0	+	+	+
**30**	15	33.9	+	+	+
**31**	15	28.1	+	+	+
**32**	15	38.6	+	+	+
**33 Uninfected**	15	-	-	-	-
**34**	20	31.0	+	+	+
**35**	20	30.5	+	+	+
**36**	20	36.6	+	+	+
**37**	20	35.8	+	+	+
**38**	20	30.0	+	+	+
**39**	20	28.8	+	+	+
**40**	20	31.9	+	+	+
**41**	20	34.5	+	+	+
**42**	20	36.6	+	+	+
**43**	20	29.3	+	+	+
**44 Uninfected**	20	-	-	-	-
**45**	25	35.6	+	+	+
**46**	25	30.6	+	+	+
**47**	25	32.5	+	+	+
**48**	25	30.0	+	+	+
**49**	25	33.2	+	+	+
**50**	25	34.3	+	+	+
**51**	25	-	-	-	-
**52**	25	28.8	+	+	+
**53**	25	30.3	+	+	+
**54**	25	29.0	+	+	+
**55 Uninfected**	25	-	-	-	-
**56 *B*. *malayi* +**	Positive Control	11.4	+	+	+
**57 NTC**	No Template Control	-	-	-	-

### Assay specificity testing

In order to verify the specificity of the “Backpack PCR”-based assay for the detection of *B*. *malayi* DNA, gDNA samples from *D*. *immitis*, *M*. *perstans*, *W*. *bancrofti*, *B*. *pahangi*, *A*. *viteae*, *and L*. *loa* were assayed along with *B*. *malayi* DNA as a positive control. To verify that these samples contained amplifiable filarial parasite DNA, extracts from all species were subjected to amplification utilizing the previously described pan-filarial primer set (DIDR) [49], and all samples amplified successfully (S1 Fig). When tested using the *B*. *malayi* field-friendly PCR platform described above, all pools containing non-Brugian filarial DNA tested negative, regardless of whether results were visualized by gel electrophoresis or test strip-based detection. However, *B*. *pahangi* genomic DNA produced positive results, indicating cross-reactivity, when examined by both visualization techniques (S1 Fig). Although the primers and probe selected for use with this assay were designed using the HhaI repeat DNA sequence from *B*. *malayi* (GenBank Accession No. AF499129.1), these results indicate that this assay also detects the closely related animal parasite *B*. *pahangi*. This is not surprising since the HhaI repeat found in *B*. *malayi* has a sequence that is 89% identical to the HhaI repeat found in *B*. *pahangi* [40]. Thus, the assay we describe here is *Brugia* genus-specific, but not *B*. *malayi* species-specific.

### Blind test for assay validation

To further validate the use of our field-friendly platform and to further demonstrate its comparable sensitivity to real-time PCR, a series of 50 mosquito samples were prepared and coded blind to the processing technician. Following NaOH-based DNA extraction, each sample underwent cPCR-based amplification using both the standard and miniPCR instruments, followed by analysis using both gel electrophoresis and test strip-based detection methodologies. Quantitative real-time PCR was also performed for comparison. For 48 out of 50 samples, results for all assays were in agreement (Table 3). Examination of the two discordant samples revealed that in one instance (Sample #13) all assays yielded negative results with the exception of standard amplification coupled with test strip-based detection. In the second instance of discordance (Sample #46), real-time PCR and test strip-based detection assays returned positive results, while the gel electrophoresis results were negative. Thus, real-time PCR and the test strip-based detection agreed on 49 of 50 samples.

**Table 3:** Comparative testing of five methods for the detection of *B*. *malayi* PCR amplification products.

Sample #	Sample Content (# of *A*. *aegypti* mosquitoes / number of L3 larvae)	Real-Time PCR Result (Ct value)	Gel Result–Standard PCR	Test-Strip Result–Standard PCR	Gel Result -miniPCR	Test-Strip Result–miniPCR
**22**	5 / 1	32	+	+	+	+
**32**	5 / 1	27.7	+	+	+	+
**38**	5 / 1	30.2	+	+	+	+
**47**	5 / 1	30.5	+	+	+	+
**2**	10 / 1	29.9	+	+	+	+
**17**	10 / 1	29	+	+	+	+
**29**	10 / 1	30.6	+	+	+	+
**37**	10 / 1	29.7	+	+	+	+
**49**	10 / 1	32.2	+	+	+	+
**9**	15 / 1	28.9	+	+	+	+
**11**	15 / 1	26.9	+	+	+	+
**18**	15 / 1	32.9	+	+	+	+
**23**	15 / 1	29.4	+	+	+	+
**1**	20 / 1	26.8	+	+	+	+
**4**	20 / 1	28	+	+	+	+
**6**	20 / 1	34.8	+	+	+	+
**25**	20 / 1	32.4	+	+	+	+
**43**	20 / 1	36.5	+	+	+	+
**45**	20 / 1	28.6	+	+	+	+
46*	20 / 1	39.8	-	+	-	+
13*	25 / 1	-	-	+	-	-
**21**	25 / 1	36.5	+	+	+	+
**30**	25 / 1	28.6	+	+	+	+
**33**	25 / 1	27.5	+	+	+	+
**35**	25 / 1	31.4	+	+	+	+
**40**	25 / 1	30.5	+	+	+	+
**41**	25 / 1	27.5	+	+	+	+
**44**	25 / 1	29.9	+	+	+	+
**3**	5 / 0	-	-	-	-	-
**5**	5 / 0	-	-	-	-	-
**7**	5 / 0	-	-	-	-	-
**8**	5 / 0	-	-	-	-	-
**10**	5 / 0	-	-	-	-	-
**42**	5 / 0	-	-	-	-	-
**12**	10 / 0	-	-	-	-	-
**19**	10 / 0	-	-	-	-	-
**20**	10 / 0	-	-	-	-	-
**28**	10 / 0	-	-	-	-	-
**50**	10 / 0	-	-	-	-	-
**14**	15 / 0	-	-	-	-	-
**15**	15 / 0	-	-	-	-	-
**24**	15 / 0	-	-	-	-	-
**27**	15 / 0	-	-	-	-	-
**39**	15 / 0	-	-	-	-	-
**48**	15 / 0	-	-	-	-	-
**26**	20 / 0	-	-	-	-	-
**34**	20 / 0	-	-	-	-	-
**36**	20 / 0	-	-	-	-	-
**16**	25 / 0	-	-	-	-	-
**31**	25 / 0	-	-	-	-	-

* Testing of sample produced a false negative result by one or more detection methods.

## Discussion

**Fig 3:**
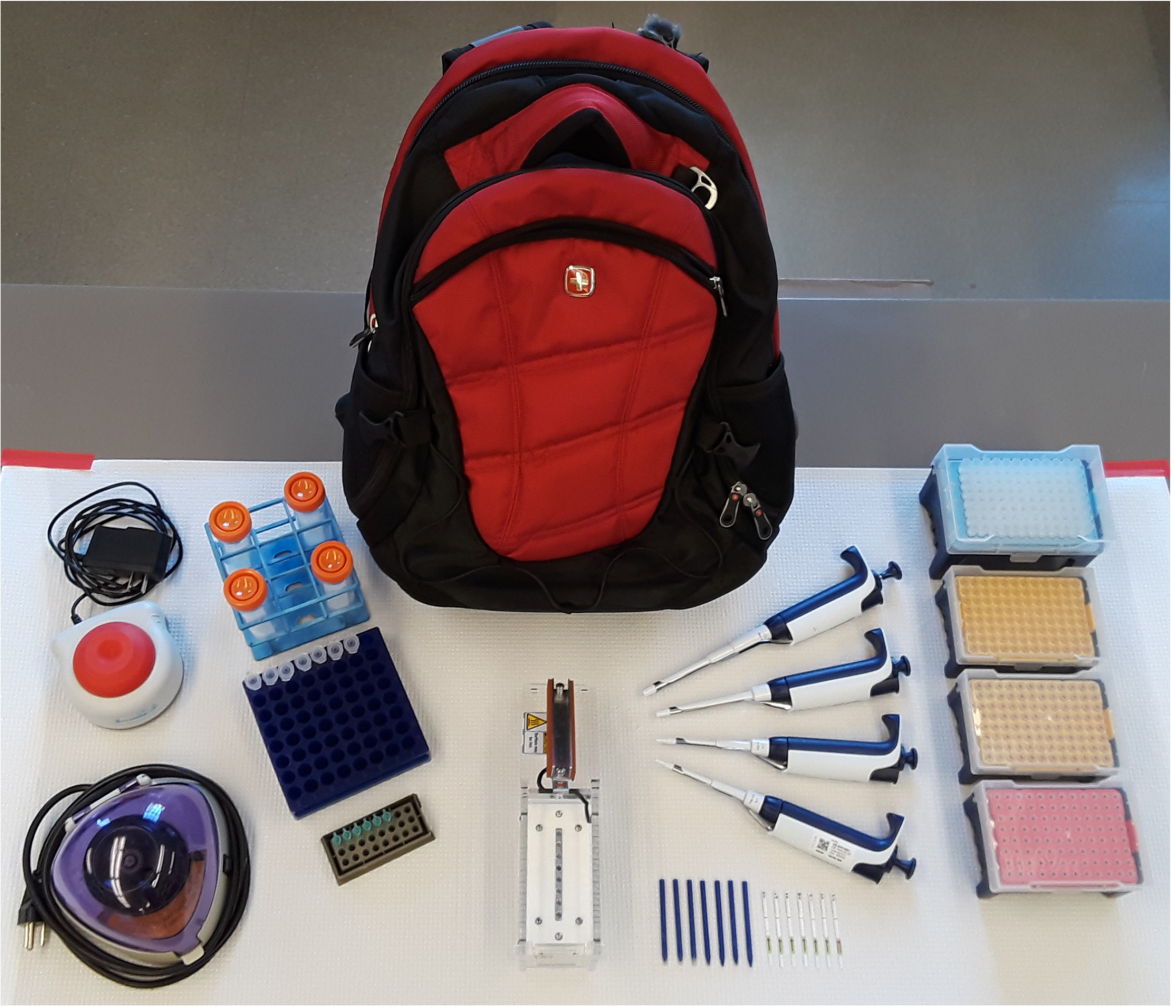
The contents of the “Backpack PCR” platform. The entire "Backpack PCR" platform weighs less than 10 lbs and can be easily transported by a single person. The “Backpack PCR” platform, which couples a simple and inexpensive NaOH-based extraction method with miniPCR amplification and test strip detection, will facilitate the use of molecular xenomonitoring in resource-limited settings. Furthermore, as this platform is based upon commercially available technologies, it will prove readily adaptable to the detection of *W*. *bancrofti* and other mosquito-borne pathogens. Research to develop this platform for the detection of other parasite and viral pathogens found in mosquitos is ongoing in our laboratory.

As a platform for filarial parasite detection, the coupling of a simple DNA extraction method with a field-friendly amplification platform and test strip-based detection technology has the potential to greatly expand the reach of MX efforts. Due to the reduced need for infrastructure and expensive, highly technical equipment, “Backpack PCR” is ideally suited for use in endemic locations currently lacking the capacity to perform real-time PCR reactions for MX purposes. We have demonstrated that this “Backpack PCR” platform has the capacity to reliably detect a single *B*. *malayi* L3 infective larva in pools of up to 25 uninfected *A*. *aegypti* mosquitoes. "Backpack PCR” minimizes equipment needs since it requires only a dry bath, a low-speed microcentrifuge, and a portable, battery-powered and smartphone-controlled thermal cycler (miniPCR) which weighs less than 1 lb. All of the equipment and materials needed for the assay can be easily transported in a backpack (Fig 3) and sample analysis can be carried out at, or near the point-of-collection in remote locations with limited resources. Building upon the proof-of-concept work described here, the development of a parallel assay for the detection of *W*. *bancrofti* will further empower local scientists by enabling their independent use of MX as a tool for the mapping of filarial parasite prevalence and for post-MDA surveillance.

Despite the many advantages of this point-of-collection platform, the current design does present some challenges. Foremost, at a cost of approximately $7.50 per pool of mosquitoes, extraction and detection using this assay requires a significantly greater reagent investment than does extraction coupled with real-time PCR testing, estimated to cost $5.45 per equally-sized pool. Much of the cost of this system is due to the expensive test-strips, so we are actively researching less expensive alternatives that could reduce the cost to be competitive with real-time PCR. Utilization of this field-friendly platform will eliminate the need to maintain and service the sophisticated thermal cyclers required to perform real-time PCR diagnostics in a laboratory setting. In addition, the large capital commitments required for the initial purchase of such real-time PCR instruments would be eliminated, substantially lowering both initial overhead costs, and the recurring costs that arise from maintenance contracts and service fees. Furthermore, in many settings, increased reagent costs could be offset by eliminating the expenses associated with transporting and/or shipping samples to a reference laboratory for analysis. Eliminating the need for shipment, oftentimes out of country, has added benefits, as government regulations in many endemic countries require samples to be tested in their country of origin.

Although not unique to MX assays [48], the capacity of this assay to amplify DNA from both the human parasite *B*. *malayi* and the animal parasite *B*. *pahangi* presents an additional limitation. While sufficient data does not exist to reliably estimate the prevalence of *B*. *pahangi* within most locations co-endemic for both Brugian parasites, this shortcoming certainly merits additional investigation in such co-endemic areas.

Effectively trapping large numbers of *Anopheles* and *Mansonia* mosquitoes, primary vectors of *B*. *malayi* [50], presents the most substantial obstacle for all *Brugia-*based xenomonitoring efforts. Historically, such trapping difficulties have greatly restricted xenomonitoring efforts in *Brugia*-endemic locations, resulting in very few published examples of implementation [47, 51]. However, novel trap designs and improved trapping techniques continue to emerge [52–56] and it is imperative that the appropriate molecular tools be available to most effectively capitalize upon trap improvements as they occur. More importantly, the successful development of the “Backpack PCR” platform described here provides proof-of-principle for the development of future MX assays for the detection of other mosquito-borne infections. Accordingly, the development of a similar “Backpack PCR”-based assay for the detection of *W*. *bancrofti*, the parasite responsible for approximately 90% of the global LF burden, would be of significant use to the research community, and efforts to create such a platform in our laboratory are currently underway. Similarly, this method could also be extended to the detection of pathogens causing other mosquito-borne infections such as malaria, Zika, dengue fever, Chikungunya and others.

## Supporting information

S1 Fig Evaluation of the specificity of detection of the “Backpack PCR” platform. (A) Amplification reactions utilizing the previously published pan-filarial DIDR primer set were conducted to demonstrate the integrity and amplifiable-nature of the isolated DNA templates from various filarial parasites. 100 bp ladder (lane 1), *B*. *malayi* (lane 2), *D*. *immitis* (lane 3), *M*. *perstans* (lane 4), *W*. *bancrofti* (lane 5), *B*. *pahangi* (lane 6), *A*. *viteae* (lane 7), *L*. *loa* (lane 8), No Template Control (lane 9), 100 bp ladder (lane 10).
(B) These same genomic DNA samples were included as template in amplification reactions utilizing the *Brugia* spp.-specific primer pair employed for test strip-based detection and amplification products were visualized on an agarose gel. 100 bp ladder (lane 1), *B*. *malayi* (lane 2), *D*. *immitis* (lane 3), *M*. *perstans* (lane 4), *W*. *bancrofti* (lane 5), *B*. *pahangi* (lane 6), *A*. *viteae* (lane 7), *L*. *loa* (lane 8), No Template Control (lane 9), 100 bp ladder (lane 10). (C) With the exception of *A*. *viteae* (product volume was exhausted) amplification products were also visualized using test strip-based detection. *B*. *malayi* (strip 1), *D*. *immitis* (strip 2), *M*. *perstans* (strip 3), *W*. *bancrofti* (strip 4), *B*. *pahangi* (strip 5), *L*. *loa* (strip 6), No template control (strip 7).
(TIF)
