# Supplementary figures and images for: Backpack PCR: A point-of-collection diagnostic platform for the rapid detection of Brugia parasites in mosquitoes
Source: PLoS Negl Trop Dis. 2018 Nov 21;12(11):e0006962. doi: 10.1371/journal.pntd.0006962 (PMC6281274; doi:10.1371/journal.pntd.0006962)

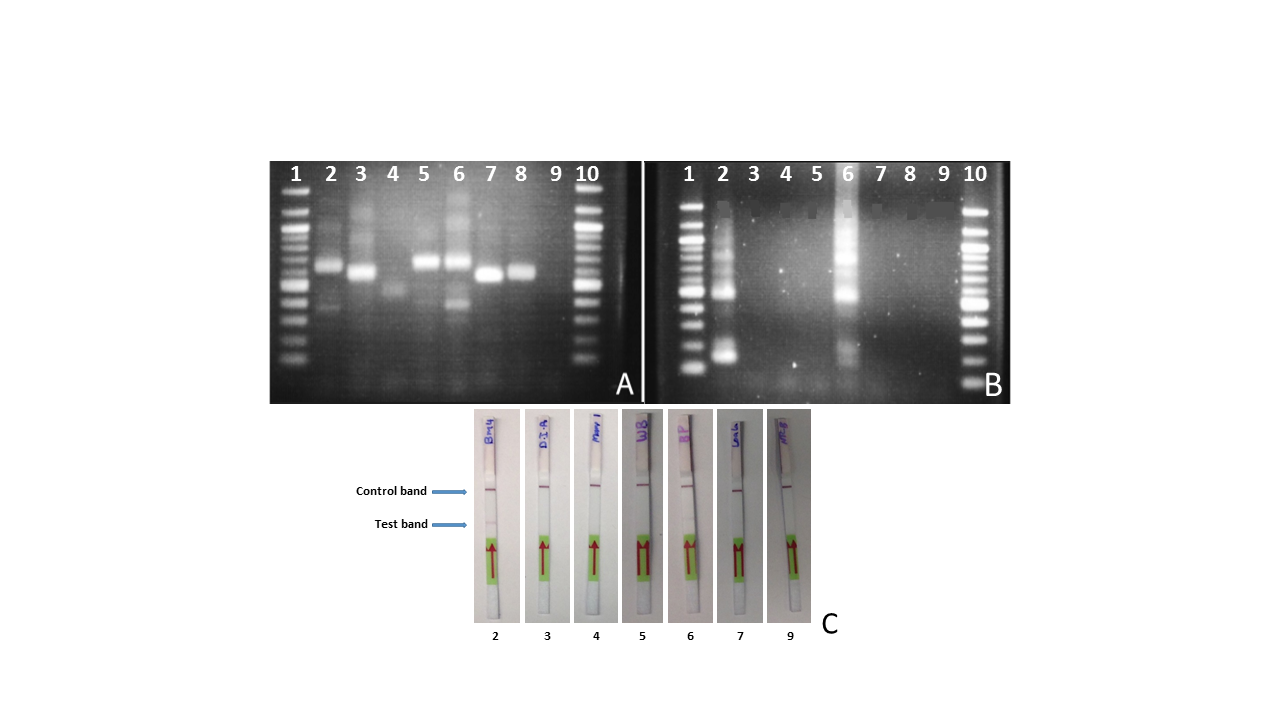

Supplement: S1 Fig — (A) Amplification reactions utilizing the previously published pan-filarial DIDR primer set were conducted to demonstrate the integrity and amplifiable-nature of the isolated DNA templates from various filarial parasites. 100 bp ladder (lane 1), B. malayi (lane 2), D. immitis (lane 3), M. perstans (lane 4), W. bancrofti (lane 5), B. pahangi (lane 6), A. viteae (lane 7), L. loa (lane 8), No Template Control (lane 9), 100 bp ladder (lane 10). (B) These same genomic DNA samples were included as template in amplification reactions utilizing the Brugia spp.-specific primer pair employed for test strip-based detection and amplification products were visualized on an agarose gel. 100 bp ladder (lane 1), B. malayi (lane 2), D. immitis (lane 3), M. perstans (lane 4), W. bancrofti (lane 5), B. pahangi (lane 6), A. viteae (lane 7), L. loa (lane 8), No Template Control (lane 9), 100 bp ladder (lane 10). (C) With the exception of A. viteae (product volume was exhausted) amplification products were also visualized using test strip-based detection. B. malayi (strip 1), D. immitis (strip 2), M. perstans (strip 3), W. bancrofti (strip 4), B. pahangi (strip 5), L. loa (strip 6), No template control (strip 7). (TIF) [file pntd.0006962.s001.tif]
